# Ravulizumab demonstrates long-term efficacy, safety and favorable patient survival in patients with paroxysmal nocturnal hemoglobinuria

**DOI:** 10.1007/s00277-025-06193-5

**Published:** 2025-01-22

**Authors:** Austin Kulasekararaj, Robert Brodsky, Hubert Schrezenmeier, Morag Griffin, Alexander Röth, Caroline Piatek, Masayo Ogawa, Ji Yu, Ami S. Patel, Yogesh Patel, Rosario Notaro, Kensuke Usuki, Alexander Kulagin, Sandra Gualandro, Wolfgang Füreder, Regis Peffault de Latour, Jeff Szer, Jong Wook Lee

**Affiliations:** 1 Department of Haematological Medicine, King’s College Hospital, National Institute for Health Research and Wellcome King’s Clinical Research Facility and King’s College London, London, UK; 2 Division of Hematology, Johns Hopkins Medicine, Baltimore, MD USA; 3 Institute of Transfusion Medicine, University of Ulm, Ulm, Germany; 4 Institute for Clinical Transfusion Medicine and Immunogenetics, German Red Cross Blood Transfusion Service Baden-Württemberg-Hessen and University Hospital Ulm, Ulm, Germany; 5 St James’s Hospital, NHS Teaching Hospitals, Leeds, UK; 6 West German Cancer Center, University Hospital Essen, University of Duisburg-Essen, Essen, Germany; 7 Jane Anne Nohl Division of Hematology and Center for the Study of Blood Diseases, USC Norris Comprehensive Cancer Center, Los Angeles, CA USA; 8 Alexion, AstraZeneca Rare Disease, Boston, MA USA; 9 Core Research Laboratory, Istituto per lo Studio, la Prevenzione e la Rete Oncologica (ISPRO), Florence, Italy; 10 Haematology, Azienda Ospedaliero Universitaria Careggi, Florence, Italy; 11 NTT Medical Center Tokyo, Tokyo, Japan; 12 RM Gorbacheva Research Institute, Pavlov University, St Petersburg, Russia; 13 Department of Hematology, University of São Paulo Medical School, São Paulo, Brazil; 14 Division of Hematology and Hemostaseology, Department of Internal Medicine I, Medical University of Vienna, Vienna, Austria; 15 Assistance Publique– Hôpitaux de Paris, Saint-Louis Hospital, Paris, France; 16 Department of Clinical Haematology, Peter MacCallum Cancer Centre and the Royal Melbourne Hospital, Melbourne, VIC Australia; 17 Division of Hematology-Oncology, Hanyang University Seoul Hospital, Seoul, Republic of Korea

**Keywords:** Complement inhibitor, Intravascular hemolysis, Lactate dehydrogenase, Paroxysmal nocturnal hemoglobinuria, Ravulizumab, Survival

## Abstract

Ravulizumab is a second-generation complement component 5 (C5) inhibitor (C5i) approved for the treatment of paroxysmal nocturnal hemoglobinuria (PNH) following positive results from two pivotal trials in patients with PNH originally naive to C5i treatment and eculizumab-experienced patients with PNH. In both trials, after the 26week primary evaluation period, all patients received ravulizumab for up to 6 years. To report ravulizumab treatment outcomes in patients with PNH originally naive to C5i treatment and eculizumab-experienced patients with PNH treated for up to 6 years. Originally C5i-naive (*N* = 244) and eculizumab-experienced (*N* = 191) patients with PNH continued ravulizumab treatment for up to 6 years. Major adverse vascular events (MAVEs; including thrombotic events [TEs]) and survival are reported, including a comparison of survival with untreated patients from the International PNH Registry. Laboratory parameters for intravascular hemolysis (IVH) are also described. For up to 6 years (1468.0 patient-years of exposure), ravulizumab provided durable control of terminal complement activity and IVH, resulting in a low incidence of MAVEs (including TEs) reported (MAVE rate: 0.7–1.4 per 100 patient-years) and, compared with untreated patients from the International PNH Registry, reduced the risk of mortality by five-fold. The few breakthrough IVH events reported (*N* = 122) were commonly associated with complement-amplifying conditions, and only two events (1.8%) were associated with suboptimal inhibition of C5 (i.e. serum free C5 ≥ 0.5 µg/mL). These results support the long-term use of ravulizumab as the first-line treatment of choice for patients with PNH. Trial registration details: NCT01374360; registered: October 29, 2004; NCT02946463; registered: October 27, 2016; NCT03056040; registered: June 05, 2017.

**Supplementary Information:**

The online version contains supplementary material available at 10.1007/s00277-025-06193-5.

## Introduction

Paroxysmal nocturnal hemoglobinuria (PNH) is a rare, chronic hematological disorder characterized by uncontrolled terminal complement activity on the surface of PNH blood cells, resulting in intravascular hemolysis (IVH), increased risk of thromboembolic events (TEs), and organ damage [1–4]. If left untreated, patients with PNH experience significant impairments in quality of life (QoL) and are at a significantly increased risk of morbidity and premature mortality [1, 3].

The terminal complement component 5 (C5) inhibitors (C5is) eculizumab (Soliris^®^, Alexion Pharmaceuticals Inc.) and ravulizumab (Ultomiris^®^, Alexion Pharmaceuticals Inc.) have revolutionized treatment outcomes for patients with PNH. Ravulizumab is a second-generation C5i engineered from eculizumab designed to achieve immediate, complete, and sustained inhibition of C5 at an extended weight-based dosing regimen (every 8 weeks [Q8W] vs. every 2 weeks [Q2W]) [5–8]. Two pivotal, long-term phase 3 clinical trials that evaluated the efficacy and safety of ravulizumab in C5i-naive (study 301; NCT02946463) [9] and eculizumab-experienced (study 302; NCT03056040) [10] patients with PNH have demonstrated the durable efficacy and tolerability of ravulizumab treatment for up to 2 years, irrespective of their prior experience with eculizumab. In addition, a lower rate of breakthrough intravascular hemolysis (BT-IVH) has been reported in patients treated with ravulizumab compared with eculizumab, owing to its improved pharmacokinetic and pharmacodynamic profile [11].

Where available, ravulizumab is the standard of care treatment for patients with PNH. Danicopan (Voydeya™, Alexion, AstraZeneca Rare Disease), a factor D inhibitor, is approved as add-on therapy to ravulizumab or eculizumab for the treatment of patients with PNH with clinically significant extravacular hemolysis [12, 13]. Other approved treatments for patients with PNH include crovalimab (PiaSky^®^, F. Hoffmann-La Roche; C5i), pegcetacoplan (Aspaveli^®^/Empaveli^®^, Apellis Pharmaceuticals; complement C3 inhibitor) and iptacopan (Fabhalta^®^, Novartis; factor B inhibitor) [14–18].

The collection of data on long-term treatment outcomes and PNH disease progression is essential, owing to the chronic life-threatening nature of the disease and uncontrolled terminal complement activation on the surface of PNH blood cells. Here, we report long-term efficacy, safety and survival outcomes with ravulizumab treatment in patients with PNH who were originally naive to C5is and eculizumab-experienced patients with PNH for up to 6 years. We also compared the effect of ravulizumab treatment on survival in patients with PNH who were originally naive to C5is with untreated patients from the International PNH Registry (NCT01374360) [19].

## Methods

### Study designs and patient populations

The designs for studies 301 and 302 have been previously described [5, 9, 10, 20, 21]. Briefly, adult (≥ 18 years of age) C5i-naive (study 301) and eculizumab-experienced (study 302) patients with a diagnosis of PNH and no history of bone marrow transplantation were recruited [9, 10]. At screening, patients in the C5i-naive group reported clinical characteristics associated with high disease activity, defined as lactate dehydrogenase (LDH) levels ≥ 1.5 × upper limit of normal (ULN; 246 U/L) and at least one sign or symptom of PNH [9]. Conversely, patients in the eculizumab-experienced group were clinically stable, with LDH levels ≤ 1.5 × ULN, and had received eculizumab at least 6 months before study entry [10]. For the primary evaluation period (26 weeks), patients were randomized 1:1 to receive either fixed doses of eculizumab (Q2W) (outcomes not included in this analysis) or weight-based ravulizumab dosing (Q8W), as previously described [9, 10]. The primary evaluation period was followed by an open-label extension (OLE) period during which patients either continued treatment with ravulizumab or switched treatment from eculizumab to ravulizumab for up to 5 years. As the objective of this study was to evaluate ravulizumab treatment outcomes, only periods of ravulizumab exposure were included in analysis. Periods of eculizumab treatment experience (i.e. the randomization period) were not included.

The International PNH Registry is a global, non-interventional study [19, 22, 23] that has prospectively collected and evaluated data in patients with PNH, including disease progression, safety, clinical outcomes, morbidity and mortality. It is the largest global registry for patients with PNH [24]. As of October 2023, 3374 patients from 28 countries from Asia, Central America, Europe, North America, Oceania and South America were enrolled in the International PNH Registry.

### Analysis endpoints

Survival analyses were performed for ravulizumab-treated patients in each study population. In addition, the survival of patients treated with ravulizumab in the C5i-naive group was compared with the survival of untreated patients from the International PNH Registry (i.e. patients with PNH with no treatment experience with complement inhibitors) with reported high disease activity (LDH level ≥ 1.5 × ULN and at least 1 sign or symptom of PNH), ≥ 5% of PNH granulocyte population and no history of bone marrow transplantation at registry enrollment (baseline). Patients in the eculizumab-experienced group were excluded from this analysis owing to prior treatment history.

Ravulizumab treatment outcomes of interest included the proportion of patients experiencing major adverse vascular events (MAVEs, including TEs), change in LDH level from baseline, the proportion of patients experiencing BT-IVH (defined as at least one new or worsening symptom or sign of IVH with LDH level ≥ 2 × ULN [eculizumab-experienced group] and after prior reduction of LDH level to < 1.5 × ULN [C5i-naive group]), transfusion avoidance (i.e. remained transfusion free and did not require transfusion according to protocol-specified guidelines) and hemoglobin (Hb) stabilization (defined as avoidance of a ≥ 2 g/dL decrease in Hb level from baseline in the absence of transfusion during that period). QoL outcomes were measured using the Functional Assessment of Chronic Illness Therapy– Fatigue (FACIT–F) scale (range, 0–52; higher scores indicate less fatigue) and European Organisation for Research and Treatment of Cancer, Quality of Life Questionnaire–Core 30 (EORTC QLQ–C30) global health status, physical functioning, and fatigue subscales (range, 0–100; higher scores for functional scales indicate better QoL; lower scores for symptom scales indicate lower symptom levels).

Safety outcomes included the prevalence of treatment-emergent adverse events (TEAEs) and serious adverse events (SAEs) during the exposure to ravulizumab, and the proportion of patients who discontinued ravulizumab treatment.

### Statistical analysis

Unadjusted and adjusted Cox proportional hazards regression analyses were employed for comparative survival analysis. Covariates comprised age at PNH diagnosis, gender, Hb concentration, estimated glomerular filtration rate (eGFR), proportion of PNH cells, transfusion history, medical history of MAVEs (including TEs), and history of or ongoing bone marrow disease at baseline (i.e. the date of registry enrollment). The adjusted model considered all baseline covariates and was built using backward selection with covariates selected for final inclusion having reached statistical significance at the 0.10 level. At a minimum, covariates for age at PNH diagnosis and gender were included in the final adjusted model.

Long-term ravulizumab efficacy and safety outcomes were compared using descriptive statistics, and outcomes reported in less than 10 patients were excluded from the analysis. The rate of BT-IVH and MAVE events adjusted by patient-years (PYs) of exposure was calculated for this analysis and was defined as: (number of events)/(number of PYs).

**Fig. 1:**
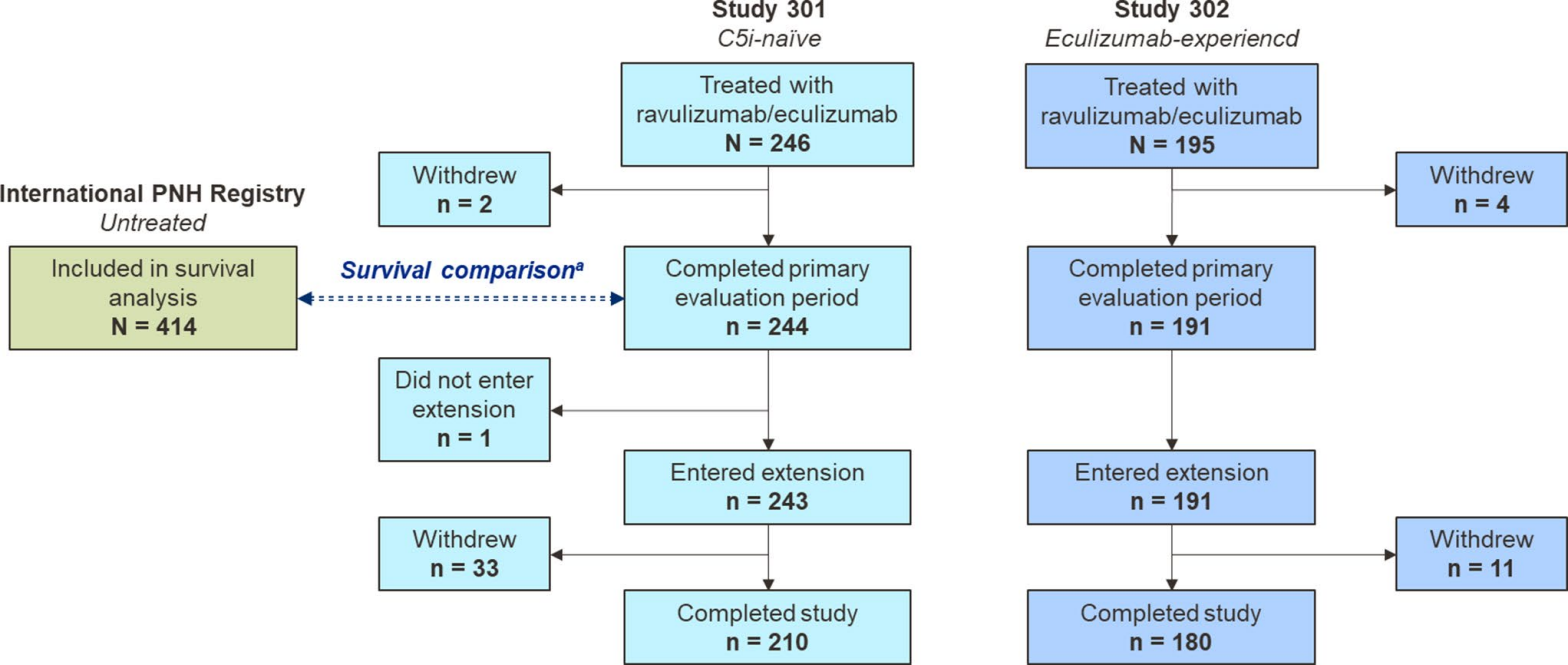
Disposition of patients included in the analyses. ^a^Overall, 243 C5i-naive patients with ≥ 5% of PNH granulocyte population were eligible for comparative survival analysis. C5i, complement component 5 inhibitor; PNH, paroxysmal nocturnal hemoglobinuria

## Results

### Patient characteristics

At study entry, 246 patients in the C5i-naive group and 195 patients in the eculizumab-experienced group received treatment with ravulizumab or eculizumab (Fig. 1). Baseline patient characteristics have been previously reported [9, 10] and are outlined in Table 1. Owing to differences in prior treatment experience with C5is (as defined in the protocol-specified inclusion criteria), the baseline clinical characteristics for PNH disease activity were different between both study populations. Overall, 244/246 (99.2%) patients in the C5i-naive group and 191/195 (97.9%) patients in the eculizumab-experienced group completed the primary evaluation period. All 191 patients in the eculizumab-experienced group and 243/244 (99.6%) patients in the C5i-naive group entered the ravulizumab OLE period.

**Table 1:** Demographics and baseline clinical characteristics of patients with PNH in the C5i-naive (study 301) and eculizumab-experienced (study 302) groups

Characteristic	C5i-naive (Study 301) *N* = 246	Eculizumab-experienced (Study 302) *N* = 195
Sex, female, n (%)	112 (45.5)	98 (50.3)
Age at PNH diagnosis, years, mean (SD)	38.8 (15.8)^a^	35.5 (14.3)^b^
Age at baseline, years, mean (SD)	45.5 (15.7)	47.7 (14.2)
**Ethnicity**,** n (%)**		
Asian	129 (52.4)	42 (21.5)
Japanese descent	34 (13.8)	12 (6.2)
Non-Japanese descent	95 (38.6)	30 (15.4)
White/Caucasian	93 (37.8)	111 (56.9)
Black/African American	6 (2.4)	8 (4.1)
American Indian/Alaska Native	2 (0.8)	0 (0.0)
Other/multiple	8 (3.3)	4 (2.1)
Not reported/unknown	8 (3.3)	30 (15.4)
Weight, kg, mean (SD)	68.7 (15.2)	72.9 (15.7)
History of MAVEs, n (%)	42 (17.1)	51 (26.2)
**Proportion of PNH cell population**,** %**,** mean (SD)**		
Total RBCs	38.6 (23.4)	60.1 (31.9)
Granulocytes	84.7 (20.0)	83.3 (22.5)
Monocytes	88.0 (16.7)	85.9 (20.0)
**eGFR**,** mL/min**,** n (%)**		
< 30	1 (0.4)	0 (0.0)
≥ 30 to < 60	27 (11.0)	15 (7.7)
≥ 60	218 (88.6)	180 (92.3)
**Proportion of PNH granulocyte population**,** %**,** n (%)**		
< 5	1 (0.4)	1 ( 0.5)
≥ 5 to < 10	2 (0.8)	3 ( 1.5)
≥ 10 to < 50	14 (5.7)	16 ( 8.2)
≥ 50	229 (93.1)	174 (89.2)
Missing	0 (0.0)	1 (0.5)
Hb, g/dL, mean (SD)^c^	9.5 (1.6)	11.0 (1.8)
LDH level, U/L, mean (SD)^d^	1606.4 (752.7)	231.6 (49.2)
History of aplastic anemia, n (%)	79 (32.1)	73 (37.4)
pRBC/whole blood transfusions ≤ 1 year before first dose, n (%)	203 (82.5)	25 (12.8)
Time on eculizumab before first study infusion, years, mean (SD)	NA	5.8 (3.5)

^a^*N* = 241; ^b^*N* = 194; ^c^Normal range, 11.5–16.0 g/dL (women) and 13.0–17.5 g/dL (men); ^d^Normal range, 120–246 U/L

C5, complement component 5; eGFR, estimated glomerular filtration rate; GPI, glycosylphosphatidylinositol; Hb, hemoglobin; LDH, lactate dehydrogenase; MAVE, major adverse vascular event; NA, not applicable; NR, not reported; PNH, paroxysmal nocturnal hemoglobinuria; pRBC, packed red blood cell; RBC, red blood cell; SD, standard deviation

In total, 243 patients in the C5i-naive group with ≥ 5% of PNH granulocyte population were eligible for comparative survival analysis with 414 untreated patients from the International PNH Registry. Demographic and clinical characteristics at baseline (C5i-naive group) and registry enrollment are reported in Table 2, and the geographic distribution of these patients are described in Supplementary Table 1. The majority of patients were residents from Europe (*n* = 346, 52.7%) and Asia (*n* = 238, 36.2%), with Russia and South Korea reporting the highest proportions of patients (18.6% and 18.0%, respectively).

**Table 2:** Demographics and baseline clinical characteristics of patients with PNH in the C5i-naive group treated with ravulizumab and untreated patients from the International PNH Registry

Characteristic	C5i-naive (Study 301) *N* = 243^*a*^	Untreated (International PNH Registry) *N* = 414
Sex, female, n (%)	111 (45.7)	188 (45.4)
Age at PNH diagnosis, years, mean (SD)	39.4 (15.8)	42.5 (18.8)^b^
Age at baseline, years, mean (SD)^c^	46.1 (15.7)	48.2 (17.6)
**Ethnicity**,** n (%)**		
Asian	127 (52.3)	128 (31.1)
White/Caucasian	92 (37.9)	272 (65.0)
Black/African descent	6 (2.5)	7 (1.7)
Native/Aboriginal	2 (0.8)	2 (0.5)
Other/multiple	16 (6.6)	3 (0.7)
Not reported	0 (0.0)	2 (0.5)
History of bone marrow disorder, n (%)^d^		
No	152 (62.6)	166 (40.1)
Yes	91 (37.4)	210 (50.7)
Unknown	0 (0.0)	38 (9.2)
History of MAVEs, n (%)	40 (16.5)	53 (12.8)
History of pRBC/whole blood transfusions, n (%)	202 (83.1)	155 (37.4)
Hb, g/dL, mean (SD)^e^	9.5 (1.5)	9.9 (2.4)^f^
**eGFR**,** mL/min**,** n (%)**^**g**^		
< 30	1 (0.4)	3 (0.8)
≥ 30 to < 60	27 (11.0)	45 (11.6)
≥ 60 to < 90	33 (13.6)	108 (27.8)
≥ 90	182 (74.9)	232 (59.8)
Proportion of PNH granulocyte population, %, mean (SD)	85.3 (19.0)	64.1 (29.5)
Proportion **of PNH granulocyte population**,** %**,** n (%)**		
≥ 5 to < 10	2 (0.8)	11 (2.7)
≥ 10 to < 50	13 (5.4)	126 (30.4)
≥ 50	228 (93.8)	277 (66.9)

^a^Overall, 243 patients of the C5i-naïve group with ≥ 5% of PNH granulocyte population were eligible for comparative survival analysis; ^b^Defined as the earliest date of PNH diagnosis, date of first PNH symptom and date of reported granulocyte clone laboratory test; ^c^Defined as age at first infusion of study drug or registry enrollment; ^d^Defined in ravulizumab-treated patients in the C5i-naive group as a report of bone marrow failure in medical history and/or aplastic anemia or myelodysplastic syndrome in PNH medical history; ^e^Normal range, 11.5–16.0 g/dL (women) and 13.0–17.5 g/dL (men); ^f^Unknown/unavailable data for 13 patients; ^g^Unknown/unavailable data for 26 untreated patients

C5, complement component 5; eGFR, estimated glomerular filtration rate; GPI, glycosylphosphatidylinositol; Hb, hemoglobin; MAVE, major adverse vascular event; PNH, paroxysmal nocturnal hemoglobinuria; pRBC, packed red blood cell; SD, standard deviation

### Survival analysis

**Fig. 2:**
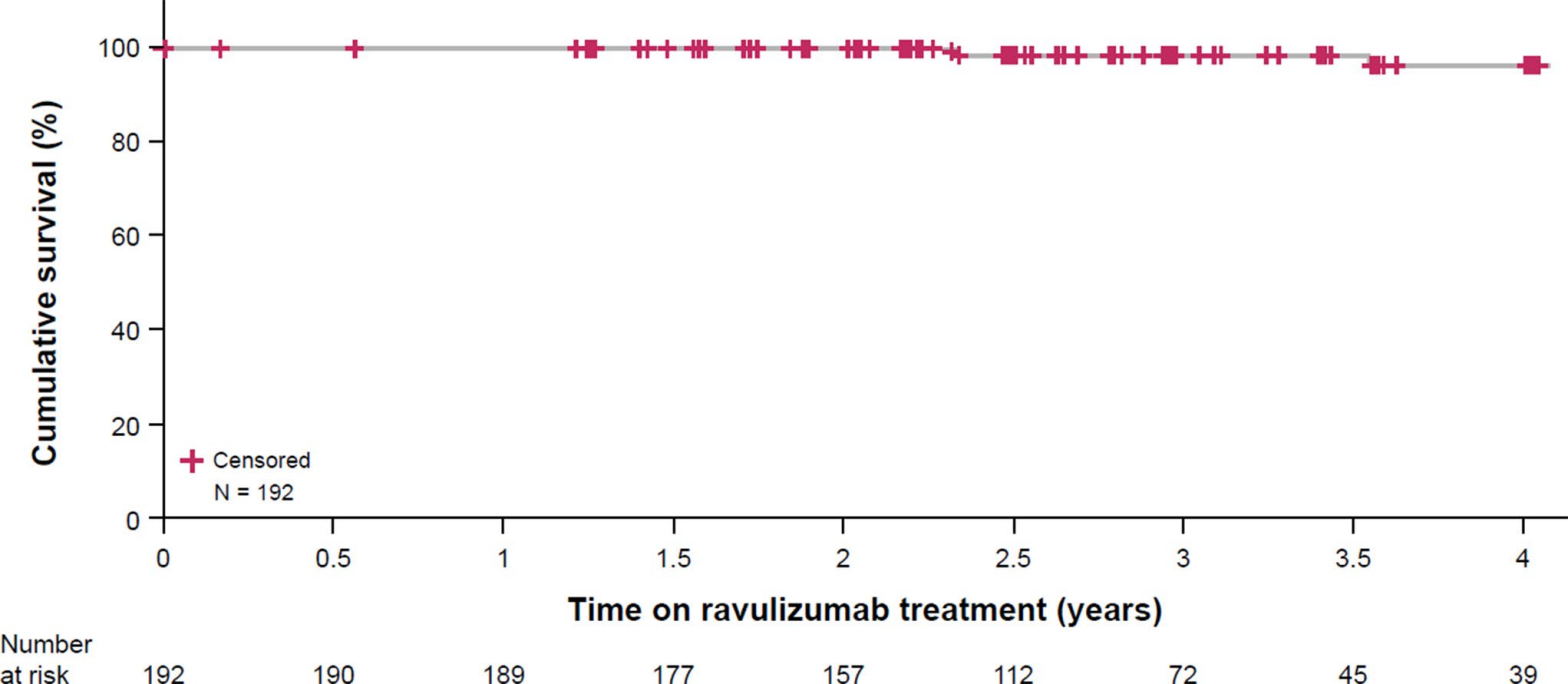
Overall survival of the eculizumab-experienced group during ravulizumab treatment. Reference: Kulasekararaj et al. [40]. Period of eculizumab treatment experience was not included in this analysis. Mortality rate = 0.6 per 100 PYs; 98.4% survival rate. PNH, paroxysmal nocturnal hemoglobinuria; PY, patient-year

**Fig. 3:**
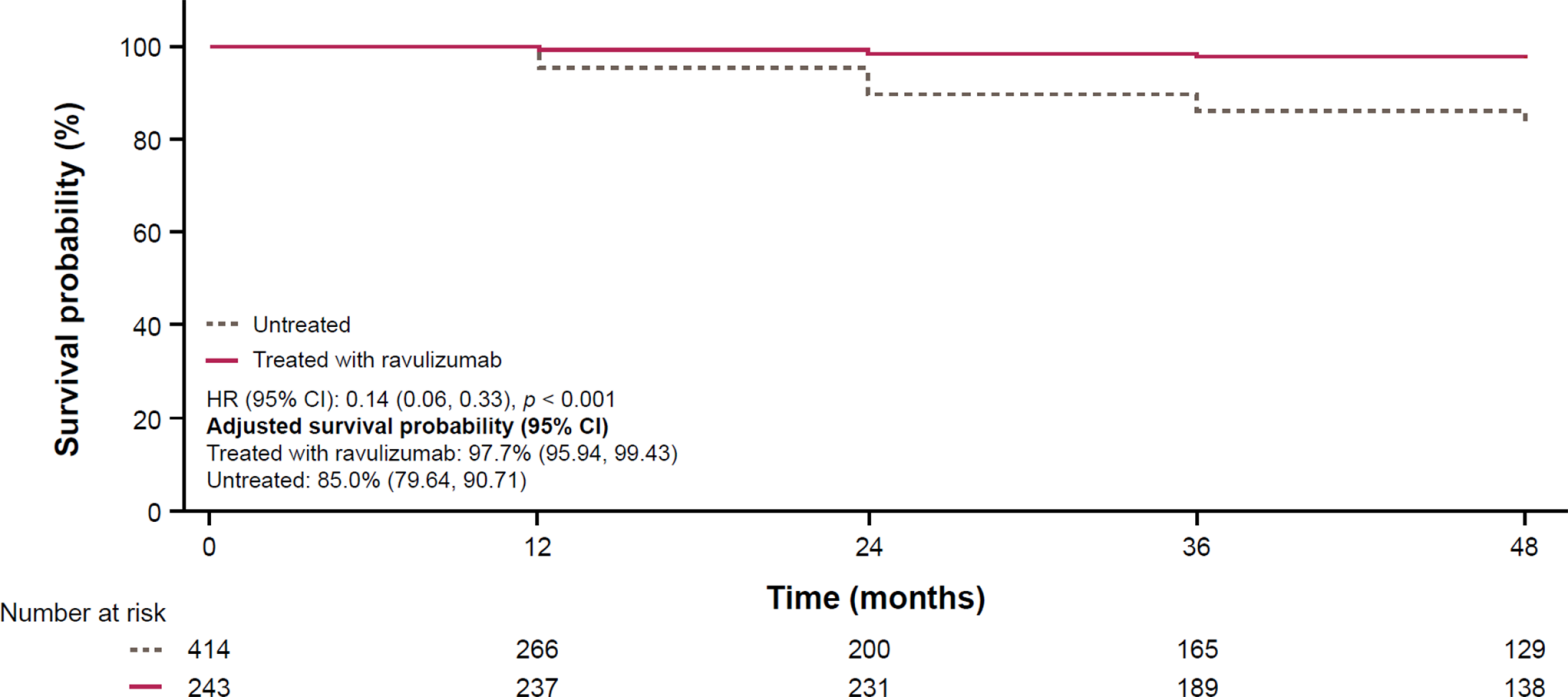
Adjusted survival analysis of ravulizumab-treated patient in the C5i-naive group and untreated patients with PNH. Reference: Kulasekararaj et al. [41]. © American Society of Hematology (2024). Reused with permission. Period of eculizumab treatment experience was not included in this analysis. Covariates included in the final adjusted model included patient transfusion history (*p* = 0.05), age at PNH diagnosis and gender. C5i, complement component 5 inhibitor; CI, confidence interval; HR, hazard ratio; PNH, paroxysmal nocturnal hemoglobinuria

Survival plots for patients treated with ravulizumab in the eculizumab-experienced and C5i-naive groups are presented in Figs. 2 and 3, respectively. Overall, no deaths were reported during the primary evaluation period. In total, 11 (2.5%; C5i-naive group, *n* = 8; eculizumab-experienced group, *n* = 3) patients died during the OLE period and an additional 3 patients in the C5i-naive group died following study withdrawal (sepsis, lung adenocarcinoma and acute myeloid leukemia). Causes of death during the study period were hypoxia due to bronchoaspiration (*n* = 1), cardiac arrest (*n* = 1), COVID-19 (*n* = 1), *Escherichia* sepsis (*n* = 1), intracranial infection (*n* = 1), meningococcal sepsis (*n* = 1; serogroup not reported), metastatic renal cancer (*n* = 1), metastatic lung cancer (*n* = 1), pulmonary sepsis (*n* = 1), sepsis (*n* = 1), and septic shock (*n* = 1). The fatality due to meningococcal sepsis (serogroup identification not performed), reported in one patient in the C5i-naive group, was the only death considered to be related to treatment (further details in Supplementary Material).

Compared with 414 untreated patients from the International PNH Registry, ravulizumab treatment in originally C5i-naive patients was significantly associated with an improved unadjusted survival probability of 86.0% at 4 years (*p* < 0.001), with a 5-fold reduced risk in mortality (mortality ratio = 0.2, 95% confidence interval [CI]: 0.09, 0.42). When adjusted for age at PNH diagnosis, gender, and transfusion history, the survival probability increases to 97.7% (95% CI: 95.94, 99.43) at 4 years (Fig. 3).

### Proportion of patients experiencing MAVEs

At baseline, 42/246 (17.1%) patients in the C5i-naive group had reported a prior medical history of at least 1 MAVE (including TEs; 54 events [rate: 3.4 per 100 PYs]). During ravulizumab treatment, MAVEs were experienced in 11/244 patients (4.5%; 13 events [rate: 1.4 per 100 PYs]). MAVEs experienced during ravulizumab treatment were deep vein thrombosis (DVT; 3 events, *n* = 3), acute myocardial infarction (1 event, *n* = 1), angina unstable (2 events, *n* = 1), cerebrovascular accident (1 event, *n* = 1), cerebral venous thrombosis (1 event, *n* = 1), coronary artery disease (1 event, *n* = 1), jugular vein thrombosis (1 event, *n* = 1), peripheral artery thrombosis (1 event, *n* = 1), and pulmonary embolism (2 events, *n* = 1).

In the eculizumab-experienced group, 51/195 (26.2%) patients had reported a prior medical history of MAVEs including TEs at baseline (87 events [rate: 3.7 per 100 PYs]). During ravulizumab treatment, MAVEs were experienced in 3/192 patients (1.6%; 4 events [rate: 0.7 per 100 PYs]). MAVEs experienced during ravulizumab treatment were cerebral infarction (*n* = 1, 2 events), DVT (*n* = 1), and thrombophlebitis (*n* = 1). No observable trends in rates of MAVEs across study periods were identified.

### Change from baseline LDH level

**Fig. 4:**
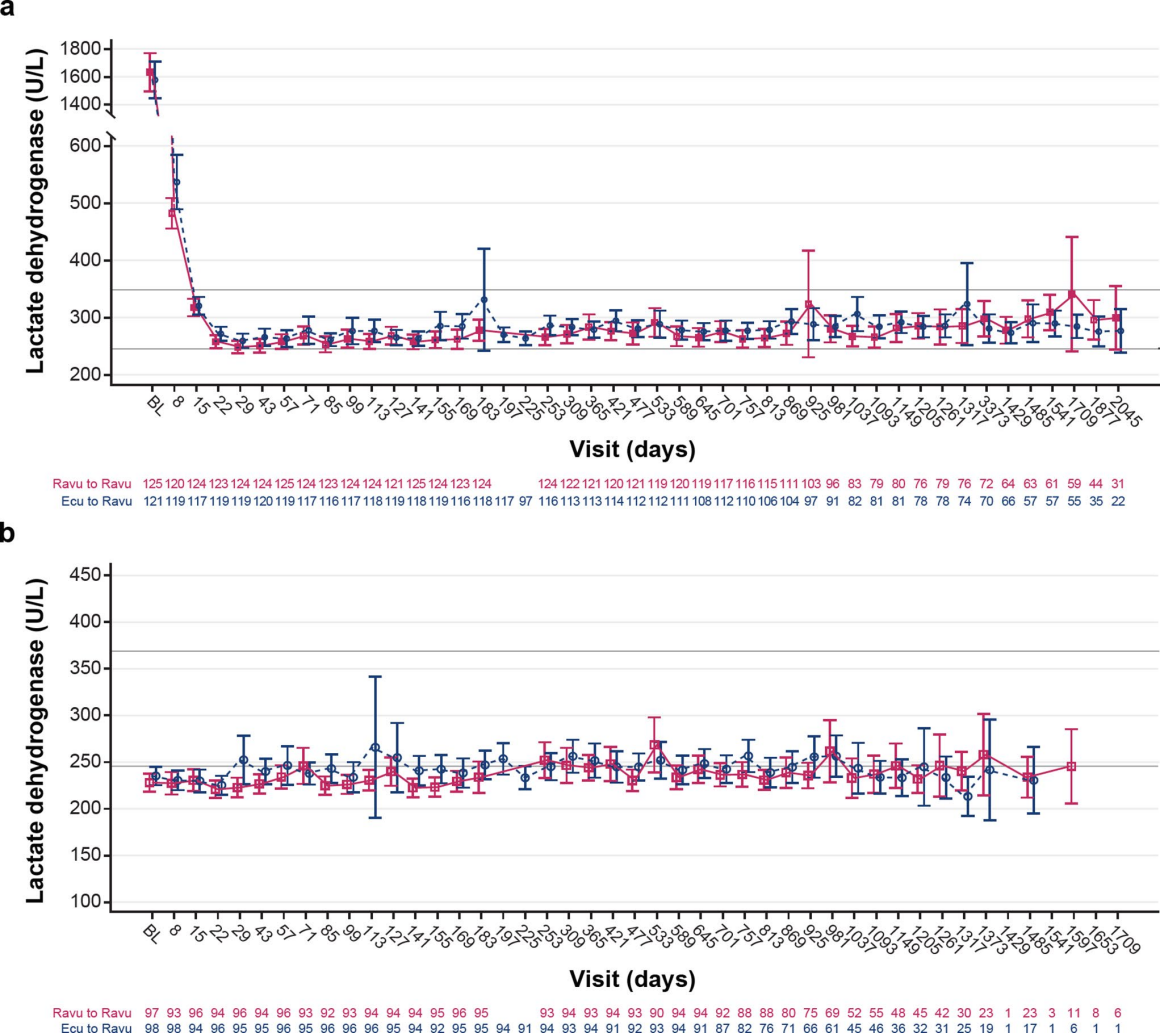
Mean (95% CI) LDH level over time for patients with PNH in (**a**) the C5i-naive group and (**b**) eculizumab-experienced group, by treatment sequence. References: **(a)** Kulasekararaj et al. [41]. © American Society of Hematology (2024). Reused with permission. **(b)** Kulasekararaj et al. [40]. Note. On both charts, the lower horizontal line indicates the ULN (246 U/L) and the upper horizontal line indicates 1.5 × ULN (369 U/L). Data points with sample sizes were less than 10 were excluded. CI, confidence interval; LDH, lactate dehydrogenase; PNH, paroxysmal nocturnal hemoglobinuria; ULN, upper limit of normal

Mean absolute change from baseline LDH levels over time is shown in Fig. 4. In the C5i-naive group, mean (standard deviation; SD) baseline LDH level was 1606.4 (752.7). Following 26 weeks of ravulizumab treatment, LDH level reduced to 277.8 (102.9) U/L, and at the end of the 6-year study period, LDH level was reported at 290.3 (127.9) U/L (an 85.4% reduction from baseline). Overall, LDH levels were reported as < 1.5 × ULN by day 15 (317.7 [85.8] U/L) and remained near normal and < 1.5 × ULN through to the end of the OLE period (Fig. 4A).

**Fig. 5:**
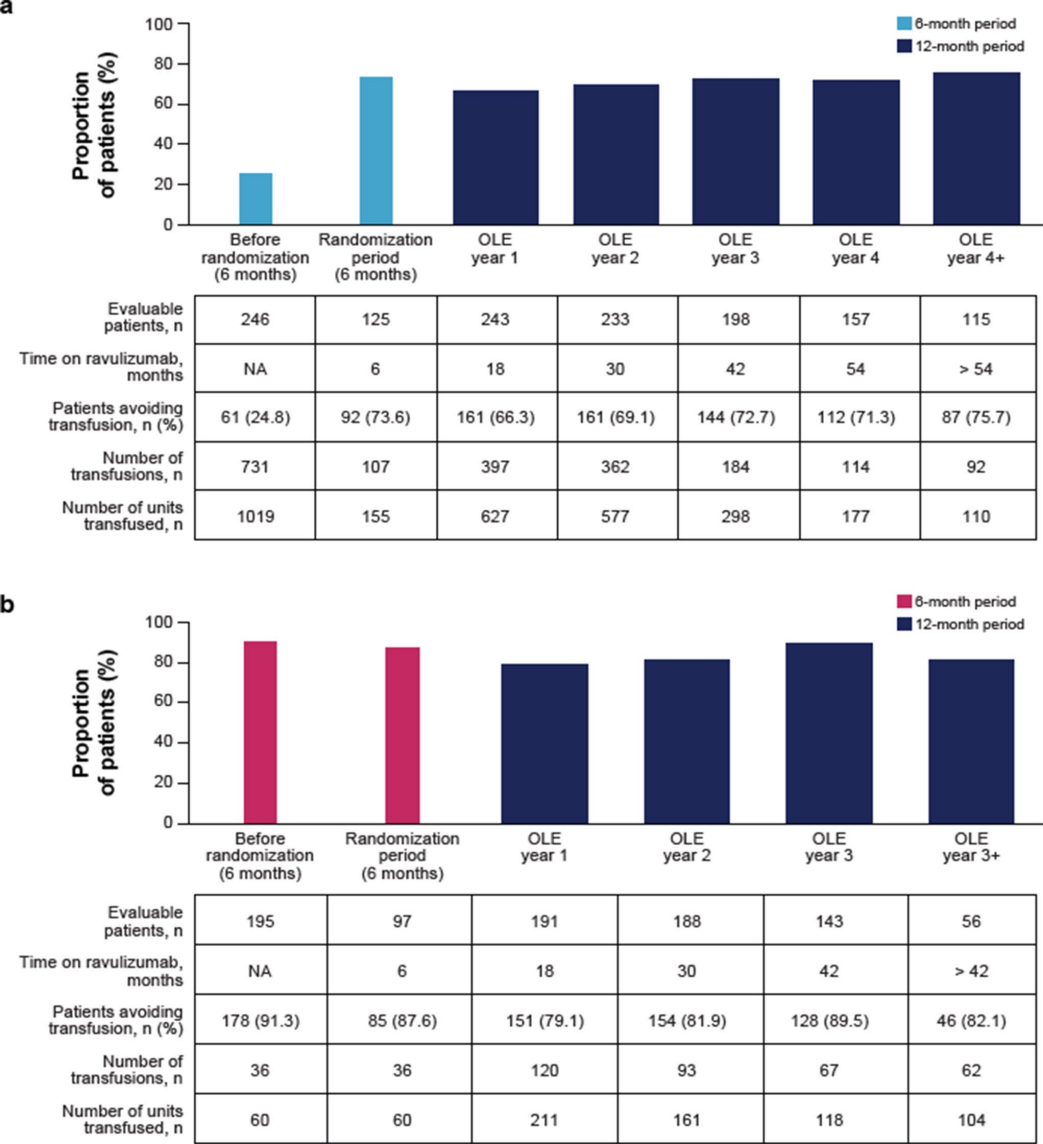
Proportion of ravulizumab-treated patients avoiding transfusion in the (**a**) C5i-naive and (**b**) eculizumab-experienced groups. References: (**a**) Kulasekararaj et al. [41]. © American Society of Hematology (2024). Reused with permission. OLE, open-label extension; NA, not applicable; PNH, paroxysmal nocturnal hemoglobinuria

Overall, in the eculizumab-experienced group, LDH levels were reported as < 1.5 × ULN throughout the study period (Fig. 4B). Baseline LDH level had been maintained at 231.6 (49.2) U/L. Minimal changes in LDH level were reported following 26 weeks of ravulizumab treatment (233.8 [51.9] U/L). Similarly, at the end of the study period (day 1597), the LDH level was near normal at 243.9 (50.6) U/L.

### Proportion of patients experiencing BT-IVH

In total, 112 BT-IVH events were reported in 36 patients in the C5i-naive group (14.8%; 94 events [rate: 1.0 per 10 PYs]) and 15 patients in the eculizumab-experienced group (7.8%; 18 events [rate: 1.0 per 30 PYs]) during ravulizumab treatment and for up to 6 years of follow-up. Overall, no cause was reported for 67/112 BT-IVH events (59.8%); 43/112 BT-IVH events (38.4%) were owing to infection or other complement-amplifying conditions (e.g. surgery or pregnancy), and 2/112 BT-IVH events (1.8%) were reported to be associated with suboptimal inhibition of C5 (i.e. serum free C5 ≥ 0.5 µg/mL).

The median (range) LDH levels at the time of BT-IVH event were 604.0 (493–4996) U/L and 603.5 (499–856) U/L for patients in the C5i-naive and eculizumab-experienced groups, respectively, and LDH levels were 2–3 × ULN during most BT-IVH events (72.3% and 77.8%, respectively). During BT-IVH events, 12.8% of patients in the C5i-naive group had LDH levels ≥ 4 × ULN, but none of the eculizumab-experienced group had an LDH level of ≥ 4 × ULN. Within the 2 weeks after a BT-IVH event, 47.3% of events (53/112) did not require a transfusion, and 50.0% (56/112) required one or two transfusions; in three events (2.7%), three or more transfusions were required. All BT-IVH events were resolved without modification to ravulizumab treatment, and none of the events reported led to discontinuation of ravulizumab or were associated with TEs.

### Proportion of patients avoiding transfusion

A total of 61/246 (24.8%) patients in the C5i-naive group and 178/195 (91.3%) patients in the eculizumab-experienced group avoided transfusions during the 6 months before randomization. Following 26 weeks of ravulizumab treatment, 73.6% of the C5i-naive group and 87.6% of the eculizumab-experienced group avoided transfusions and this was maintained in 53.9% of the C5i-naive groupand 70.7% of the eculizumabexperienced group through to the end of both ravulizumab OLEs (≥ 4 and ≥ 3 years, respectively; Fig. 5). In addition, in the C5i-naive group, the number of units transfused reduced from 1019 in the 6 months before randomization (mean [SD] number of transfusions per patient = 5.5 [4.2]) to 110 in the final 12 months of the ravulizumab OLE (mean [SD] number of transfusions per patient = 4.8 [4.8]). In the eculizumab-experienced group, 60 units were transfused in the 6 months before randomization (mean [SD] number of transfusions per patient = 3.5 [3.6]), with 104 units transfused in the final 12 months of the ravulizumab OLE (mean [SD] number of transfusions per patient = 10.4 [17.2]).

### Proportion of patients with stabilized Hb

At the end of the primary evaluation period, 163 (66.3%) patients in the C5i-naive group and 148 (75.9%) patients in the eculizumab-experienced group achieved Hb stabilization. The proportion of patients with stabilized Hb was maintained in at least 63% (C5i-naive) and at least 75% (eculizumab-experienced) of patients through each year of the OLE period.

### Change from baseline QoL

At baseline, mean (SD) FACIT–F scores were 36.8 (10.0) and 41.6 (9.5) in the C5i-naive and eculizumab-experienced groups, respectively, which increased to 44.1 (7.9) and 44.1 (8.5) by the end of the primary evaluation period. Minor changes in score were reported during the ravulizumab OLE, with 60.8% (C5i-naive) and 14.8% (eculizumab-experienced) of patients reporting at least a 5-point improvement over baseline at the end of the ravulizumab OLE.

In the C5i-naive group, mean (SD) baseline EORTC QLQ–C30 global health status, physical functioning and fatigue subscale scores were 56.8 (20.3), 76.5 (17.3), and 38.3 (23.1), which improved with ravulizumab treatment to 69.5 (20.1), 89.8 (13.4), and 19.1 (21.3) at the end of the primary evaluation period. At the end of the ravulizumab OLE, for each subscale, 52.7%, 47.3%, and 59.5% of patients reported at least a 10-point improvement over baseline score. In the eculizumab-experienced group, the respective baseline subscale scores were 72.3 (17.1), 88.0 (16.0), and 25.8 (22.1), which improved with ravulizumab treatment to 76.6 (15.6), 91.5 (15.8), and 20.2 (20.7) at the end of the primary evaluation period. At the end of the ravulizumab OLE, 29.6%, 11.1%, and 44.4% of patients reported at least a 10-point improvement over baseline score in each respective subscale.

**Table 3:** Summary of adverse events during ravulizumab treatment

	C5i-naive (Study 301) *N* = 244		Eculizumab-experienced (Study 302) *N* = 192		Total *N* = 436	
	*n* (%)	E	*n* (%)	E	*n* (%)	E
Total patient-years of exposure	NA	925.7	NA	542.3	NA	1468.0
Any TEAEs	235 (96.3)	2875	187 (97.4)	2121	422 (96.8)	4996
**TEAE by relationship to study drug**						
Related	94 (38.5)	302	60 (31.3)	224	154 (35.3)	526
Unrelated	232 (95.1)	2573	186 (96.9)	1897	418 (95.9)	4470
**TEAE by toxicity**						
Grade 1	218 (89.3)	1533	177 (92.2)	1417	395 (90.6)	2950
Grade 2	202 (82.8)	1028	147 (76.6)	566	349 (80.0)	1594
Grade 3	98 (40.2)	258	69 (35.9)	123	167 (38.3)	381
Grade 4	28 (11.5)	47	7 (3.6)	9	35 (8.0)	56
Grade 5	9 (3.7)	9	2 (1.0)	2	11 (2.5)	11
TEAE leading to study drug interruption	8 (3.3)	12	1 (0.5)	1	9 (2.1)	13
TEAE leading to study drug discontinuation	7 (2.9)	7	1 (0.5)	1	8 (1.8)	8
TEAE considered as a MAVE	11 (4.5)	13	3 (1.6)	4	14 (3.2)	17
**TEAE of special interest** ^**a**^	**73 (29.9)**	**120**	**45 (23.4)**	**66**	**118 (27.1)**	**186**
Other serious infection	34 (13.9)	48	21 (10.9)	24	55 (12.6)	72
Infusion reaction	31 (12.7)	44	27 (14.1)	40	58 (13.3)	84
Angioedema	10 (4.1)	13	2 (1.0)	2	12 (2.8)	15
Cardiac disorder	7 (2.9)	8	NR	NR	7 (1.6)	8
Sepsis	6 (2.5)	6	1 (0.5)	2	7 (1.6)	8
Meningococcal infection	1 (0.4)	1	NR	NR	1 (0.2)	1
Any treatment-emergent SAE	97 (39.8)	201	60 (31.3)	88	157 (36.0)	289
**SAE by relationship to study drug**						
Related	18 (7.4)	29	4 (2.1)	7	22 (5.0)	36
Unrelated	86 (35.2)	172	57 (29.7)	81	143 (32.8)	253
SAE leading to study drug interruption	2 (0.8)	2	0 (0)	0	2 (0.5)	2
SAE leading to study drug discontinuation	6 (2.5)	6	1 (0.5)	1	7 (1.6)	7
Death	8 (3.3)	NA	3 (1.6)	NA	11 (2.5)	NA

^a^Patients may have experienced more than one TEAE of special interest; therefore, the breakdown of events does not sum to the total number TEAEs of special interest

E, event; MAVE, major adverse vascular event; NA, not applicable; SAE, serious adverse event; TEAE, treatment-emergent adverse event

### Safety endpoints

The median duration of ravulizumab treatment was 2439.5 days (1468.0 PYs of exposure). Overall, 422 (96.8%) patients reported TEAEs during ravulizumab treatment, of which 154 patients (35.3%) experienced treatment-related TEAEs (Table 3). The five most commonly reported TEAEs (in ≥ 10.0% of patients) were headache (*n* = 130; 29.8%), upper respiratory tract infection (*n* = 113; 25.9%), nasopharyngitis (*n* = 104; 23.9%), pyrexia (*n* = 88; 20.2%), and fatigue (*n* = 61; 14.0%). One TEAE of meningococcal sepsis was reported. SAEs were reported in 157 (36.0%) patients during ravulizumab treatment, of which, 22 (5.0%) patients experienced SAEs that were deemed treatment-related.

There were no adverse events (AEs) that led to discontinuation (i.e. cessation of treatment and study withdrawal) during ravulizumab treatment in the primary evaluation period. During the ravulizumab OLE, 9 (2.1%) patients discontinued treatment owing to an TEAE. AEs leading to discontinuation of ravulizumab were myelodysplastic syndrome (*n* = 3), sepsis (*n* = 2), acute myeloid leukemia (*n* = 1), cerebrovascular accident (*n* = 1), lung adenocarcinoma (*n* = 1), and metastatic lung cancer (*n* = 1). None of the reported AEs that led to discontinuation were deemed related to ravulizumab treatment.

## Discussion

With up to 6 years of treatment follow-up in over 400 patients with PNH (1468.0 PYs of exposure), this publication reports the longest and largest data set of ravulizumab treatment outcomes in patients with PNH and is only surpassed by the data available for eculizumab in terms of length of exposure. Overall, ravulizumab provided durable control of terminal complement activity and IVH in patients with PNH who were either originally naive to C5is or had treatment experience with eculizumab (as evidenced by the maintenance of LDH levels ≤ 1.5 × ULN in the majority of patients throughout the study period), with favorable survival and no new safety signals reported. In this context, ravulizumab is a well-established and long-term proven treatment to control life-threatening consequences associated with uncontrolled terminal complement activity and IVH.

In PNH, uncontrolled terminal complement activation is associated with poor patient survival, with a 10-year survival rate of 76% reported in untreated patients with PNH [25]. TEs are the most common cause of mortality in untreated patients with PNH and have been reported to account for approximately 40–67% of deaths with a known causality [26]. In addition, patients with a history of TEs are estimated to have a ≥ 4-fold increased risk in mortality [27]. Despite a proportion (21.1%) of patients in the C5i-naive and eculizumab-experienced groups who reported a history of at least 1 MAVE (including TEs) prior to study entry (MAVE rate: 3.4 per 100 PYs and 3.7 per 100 PYs, respectively), the MAVEs rate (including TEs) during ravulizumab treatment was well controlled, considered low throughout the study (1.4 per 100 PYs and 0.7 per 100 PYs, respectively), and numerically lower than those reported in a recent real-world analysis of patients with PNH treated with complement inhibitors (2.1 per 100 PY over 2043 PYs) [28]. There was also a 5-fold reduced risk in mortality compared with untreated patients of the International PNH Registry (mortality ratio = 0.2; *p* < 0.001).

The impact of PNH on QoL is severe [29]. After 26 weeks of ravulizumab treatment, the proportion of patients with transfusion avoidance was high (> 70%) in both C5i-naive and eculizumab-experienced groups, with the proportion of those avoiding transfusion maintained to the end of the ravulizumab OLE (≥ 3 and ≥ 4 years, respectively). Moreover, for both C5i-naive and eculizumab-experienced group, including those with transfusions or non-stabilized Hb, treatment with C5i resulted in clinically meaningful improvements in FACIT–F and EORTC QLQ–C30 scores, that were maintained with ravulizumab for up to 6 years [30–33] and were in line with population normative values [34, 35].

In patients with PNH, BT-IVH can occur owing to complement-amplifying conditions such as infection, surgery, or pregnancy [36, 37]. In both C5i-naive and eculizumab-experienced groups, the rate of BT-IVH events was low (1.0 per 10 PYs and 1.0 per 30 PYs, respectively), and only 2 (1.8%) events were reported to be associated with suboptimal inhibition of C5, while 43 events (38.4%) were associated with the occurrence of complement-amplifying conditions. Therefore, our findings suggest that ravulizumab provided effective long-term terminal complement inhibition. In addition, the reported BT-IVH events were generally associated with a lower excursion of LDH level (2–3 × ULN), and so may be considered less severe than those observed with proximal monotherapy [38, 39], not associated with TEs, and did not require modification, interruption, or withdrawal of ravulizumab treatment.

As expected, owing to the length of the ravulizumab treatment follow-up (median ravulizumab treatment duration: C5i-naive group, 1423.0 days; eculizumab-experienced group, 968.0 days), the majority of included patients (95%) reported TEAEs during ravulizumab treatment, with most TEAEs deemed unrelated to ravulizumab treatment. In addition, the incidence of SAEs that were considered treatment-related was low (5.0%), and only one of the deaths reported (0.2%) was related to ravulizumab treatment. Finally, very few patients (2.1%) discontinued treatment owing to AEs, further supporting the long-term tolerability profile of ravulizumab.

There are some limitations to the current study. Regarding the comparative survival analysis, the untreated cohort of patients with PNH were recruited from the International PNH Registry, which included several countries where eculizumab is approved and had a higher proportion of patients with a PNH granulocyte population < 50% compared with patients in the C5i-naive group, suggesting differences in the two PNH populations (i.e. a greater proportion of patients in the International PNH Registry with PNH with marrow hypoplasia compared with patients in the C5i-naive group). In addition, owing to the observational nature of the PNH Registry data, complete information on cause of death was not available for untreated patients. Despite these limitations, the survival analysis adds to the body of evidence supporting the long-term benefit of ravulizumab treatment. Readers should interpret the data with consideration of the strengths and limitations of the analysis.

For up to 6 years, treatment with ravulizumab demonstrated durable control of PNH disease activity, as evidenced by the long-term maintenance of LDH levels and low rates of BT-IVH events reported, with low incidence of life-threatening AEs such as MAVEs (including TEs) and favorable patient survival. These long-term results further add to the body of evidence supporting the use of ravulizumab as the first-line, long-term treatment of choice for patients with PNH, where available, offering patients with PNH immediate, complete, and sustained inhibition of terminal complement-mediated activity.

## Electronic supplementary material

Below is the link to the electronic supplementary material.

Supplementary Material 1

## Data Availability

Alexion, AstraZeneca Rare Disease will consider requests for disclosure of clinical study participant-level data provided that participant privacy is assured through methods such as data de-identification, pseudonymization, or anonymization (as required by applicable law), and if such disclosure was included in the relevant study informed consent form or similar documentation. Qualified academic investigators may request participant-level clinical data and supporting documents (statistical analysis plan and protocol) pertaining to Alexion-sponsored studies. Further details regarding data availability and instructions for requesting information are available in the Alexion Clinical Trials Disclosure and Transparency Policy at http://alexion.com/our-research/research-and-development. Link to data request form: https://alexion.com/contact-alexion/medical-information.
