# Supplementary material for: Ravulizumab demonstrates long-term efficacy, safety and favorable patient survival in patients with paroxysmal nocturnal hemoglobinuria
Source: Ann Hematol. 2025 Jan 22;104(1):81–94. doi: 10.1007/s00277-025-06193-5 (PMC11868214; doi:10.1007/s00277-025-06193-5)
Supplement: Supplementary file 1 — Supplementary Material 1 [file 277_2025_6193_MOESM1_ESM.docx]

# Supplementary materials

**Supplementary Table 1. Geographic disposition of ravulizumab-treated patients in the C5i-naive group and untreated patients from the International PNH Registry**

| **Country and geographic region, n (%)** | **C5i-naive**  ***N = 243^a^*** | **Untreated**  ***N = 414*** | **Total**  ***N = 657*** |
| --- | --- | --- | --- |
| **Europe** | **91 (37.4)** | **255 (61.6)** | **346 (52.7)** |
| Russia | 41 (16.9) | 81 (19.6) | 122 (18.6) |
| Germany | 4 (1.6) | 58 (14.0) | 62 (9.4) |
| UK | 5 (2.1) | 36 (8.7) | 41 (6.2) |
| Spain | 5 (2.1) | 16 (3.9) | 21 (3.2) |
| France | 9 (3.7) | 8 (1.9) | 17 (2.6) |
| Netherlands | 0 (0.0) | 15 (3.6) | 15 (2.3) |
| Czech Republic | 2 (0.8) | 8 (1.9) | 10 (1.5) |
| Austria | 6 (2.5) | 2 (0.5) | 8 (1.2) |
| Italy | 8 (3.3) | 0 (0.0) | 8 (1.2) |
| Belgium | 2 (0.8) | 4 (1.0) | 6 (0.9) |
| Sweden | 1 (0.4) | 5 (1.2) | 6 (0.9) |
| Denmark | 0 (0.0) | 5 (1.2) | 5 (0.8) |
| Finland | 0 (0.0) | 5 (1.2) | 5 (0.8) |
| Poland | 5 (2.1) | 0 (0.0) | 5 (0.8) |
| Portugal | 0 (0.0) | 5 (1.2) | 5 (0.8) |
| Turkey | 1 (0.4) | 4 (1.0) | 5 (0.8) |
| Greece | 0 (0.0) | 3 (0.7) | 3 (0.5) |
| Estonia | 2 (0.8) | 0 (0.0) | 2 (0.3) |
| **Asia** | **120 (49.4)** | **118 (28.5)** | **238 (36.2)** |
| South Korea | 39 (16.0) | 79 (19.1) | 118 (18.0) |
| Japan | 33 (13.6) | 13 (3.1) | 46 (7.0) |
| Malaysia | 30 (12.3) | 4 (1.0) | 34 (5.2) |
| Taiwan | 8 (3.3) | 17 (4.1) | 25 (3.8) |
| Thailand | 8 (3.3) | 2 (0.5) | 10 (1.5) |
| Singapore | 2 (0.8) | 1 (0.2) | 3 (0.5) |
| Hong Kong | 0 (0.0) | 1 (0.2) | 1 (0.2) |
| Israel | 0 (0.0) | 1 (0.2) | 1 (0.2) |
| **North America** | **9 (3.7)** | **29 (7.0)** | **38 (5.8)** |
| USA | 5 (2.1) | 20 (4.8) | 25 (3.8) |
| Canada | 4 (1.6) | 9 (2.2) | 13 (2.0) |
| **Central/South America** | **22 (9.1)** | **2 (0.5)** | **24 (3.7)** |
| Brazil | 16 (6.6) | 0 (0.0) | 16 (2.4) |
| Argentina | 4 (1.6) | 2 (0.5) | 6 (0.9) |
| Mexico | 2 (0.8) | 0 (0.0) | 2 (0.3) |
| **Australia/New Zealand** | **1 (0.4)** | **10 (2.4)** | **11 (1.7)** |
| Australia | 1 (0.4) | 9 (2.2) | 10 (1.5) |
| New Zealand | 0 (0.0) | 1 (0.2) | 1 (0.2) |

^a^Overall, 243 patients in the C5i-naive group with ≥ 5% of PNH granulocyte population were eligible for comparative survival analysis.

PNH, paroxysmal nocturnal hemoglobinuria.

## Narrative of death in the originally C5i-naive patient

On day 840, while on a regimen of ravulizumab 3300 mg IV Q8W, the patient experienced fever with body temperature of 38.9°C, chills, vomiting, malaise, and myalgia and was later diagnosed with meningococcal sepsis. On day 842, the patient was hospitalized and was placed in the intensive care unit. The patient had previouly received the Menactra^®^ meningococcal vaccine A/C/Y/W. It was noted that the patient had a risk factor for meningococcal infection. The investigator also confirmed that the patient was not on an antibiotic prophylaxis but had no previous history of meningococcal infection. On day 843, an aerobic blood culture revealed growth of *Neisseria meningitidis.* A meningococcal serotype identification was not performed. On day 853, the patient died owing to the serious event of meningococcal sepsis. The last dose of ravulizumab was administered on day 806.

C5i, complement component 5 inhibitor; IV, intravenous; Q8W, every 8 weeks.

## Plain language summary of “Ravulizumab demonstrates long‑term efficacy, safety and favorable patient survival in patients with paroxysmal nocturnal hemoglobinuria”

### Why was this research performed?

Investigators carried out this research to understand the long-term efficacy and safety of ravulizumab, a drug approved for the treatment of paroxysmal nocturnal hemoglobinuria (PNH). PNH is a rare, chronic, progressive and potentially life-threatening blood disorder characterized by red blood cell (RBC) destruction within the blood vessels (also known as intravascular hemolysis, or IVH) and white blood cell and platelet activation, which may lead to blood clots (thrombosis). There is already a lot of information that shows that ravulizumab improves symptoms and outcomes for people with PNH (more information can be found at the end of this summary). This research aimed to understand how well ravulizumab performs over a long period of time (up to 6 years). Investigators also compared survival between people with PNH who were treated with ravulizumab and people with PNH who have never received treatment with ravulizumab or any other treatments of its type.

### How was this research performed?

To understand efficacy and safety of long-term treatment of PNH with ravulizumab, researchers followed over 400 people with PNH from two clinical trials who received ravulizumab for up to 6 years. Some of these people were new to this type of treatment, and others had been treated with a similar drug (called eculizumab) before switching to ravulizumab. Over the 6-year period, researchers monitored PNH symptoms (including signs of RBC destruction and IVH) and any serious side-effects (including major adverse vascular events such as blood clots). Survival was also compared between approximately 250 people with PNH who received ravulizumab for the first time as part of the clinical trial and more than 400 people with PNH from the International PNH Registry, a global database which collects information on people with PNH during their routine care.

### What were the findings of this research?

Treatment with ravulizumab controlled PNH disease activity and symptoms linked to PNH, which was maintained for up to 6 years. Throughout the 6-year period, measurements of RBC destruction were controlled low enough to limit the life-threatening problems linked to PNH, including the number and rate of blood clots (and other major adverse vascular events) and IVH events. Compared with untreated people with PNH, treatment with ravulizumab was linked with a 5-fold reduced risk of death. Finally, an acceptable number of serious side-effects associated with ravulizumab treatment were reported and occurred in only 5% of patients over the 6-year period.

### What are the implications of this research?

The results of this analysis suggest that long-term treatment with ravulizumab is effective in controlling PNH and the symptoms that people with PNH experience, with a favorable safety profile. Aside from eculizumab, this study is the longest assessment of treatment outcomes for any treatment for PNH and provides confidence in treating people with PNH with ravulizumab in the long term.

### Where can I find more information?

For more information on this study, you can refer to the clinical trials included in this publication (NCT02946463 and NCT03056040) or the International PNH Registry (NCT01374360).

### Key words to know

| **Complement component** | - A protein involved in the complement system |
| --- | --- |
| **Complement system** | - A part of the immune system and is essential to the body’s defense against infection - When the system is thrown out of balance, or dysregulated, these proteins can trigger a dangerous, uncontrolled cascade of reactions that attack cells and tissues resulting in harmful inflammation and the destruction of healthy cells |
| **Eculizumab and ravulizumab** | - Eculizumab (also called SOLIRIS^®^) and ravulizumab (also called ULTOMIRIS^®^) are approved treatments for people with PNH - Both treatments are types of monoclonal antibodies known as complement component 5 inhibitors which bind to the protein C5 in the blood - Both treatments are given to the patient through the vein. With eculizumab, the time to retreatment is every 2 weeks, and the time to retreatment with ravulizumab is every 8 weeks |
| **Intravascular hemolysis** | - Red blood cell destruction within blood vessels |
| **Major adverse vascular events** | - Side effects that affect the blood vessels, for example, deep vein thrombosis, myocardial infarction (heart attack), angina (chest pain) and cerebrovascular accident |
| **Monoclonal antibody** | - A protein that binds to specific targets in the body |
| **Paroxysmal nocturnal hemoglobinuria** | - A rare, chronic, progressive and potentially life-threatening blood disorder characterized by red blood cells destruction within the blood vessels and white blood cell and platelet activation, which may lead to blood clots |
| **Thrombosis** | - Formation of blood clots |
